# Physical activity and sedentary behaviour of male adolescents in Indonesia during the COVID-19 pandemic: a mixed-method case study using accelerometers, automated wearable cameras, diaries, and interviews

**DOI:** 10.1186/s44167-022-00014-0

**Published:** 2023-03-01

**Authors:** Fitria Dwi Andriyani, Katrien De Cocker, Aprida Agung Priambadha, Stuart J. H. Biddle

**Affiliations:** 1 Physically Active Lifestyles Research Group (USQ PALs), Centre for Health Research, University of Southern Queensland, Education City, 37 Sinnathamby Boulevard, Springfield Central, QLD 4300 Australia; 2 Department of Sports Education, Faculty of Sports Science, Yogyakarta State University, Yogyakarta, 55281 Indonesia; 3 Department of Movement and Sports Sciences, Faculty of Medicine and Health Sciences, Ghent University, B9000 Ghent, Belgium; 4 Department of Primary Teacher Education, Faculty of Teacher Training and Education, Ahmad Dahlan University, Yogyakarta, 55191 Indonesia

**Keywords:** LMICs, Screen time, Automated wearable camera, Accelerometers, Male adolescents, Youth

## Abstract

**Background:**

Previous physical activity and sedentary behaviour studies during the pandemic have largely utilized online surveys, with known limitations including recall bias. Employing both device-based and self-reported measurements may provide a more comprehensive picture of both behaviours. Physical activity and sedentary behaviour research in adolescents is still limited in low- and middle-income countries (LMICs), including Indonesia. Male adolescents had been identified as more active than females but have had a greater decrease in physical activity during the pandemic. The present study aimed to investigate the quantity, temporal patterns, contexts, and biopsychosocial factors of physical activity and sedentary behaviour during the COVID-19 pandemic in a small group of male Indonesian adolescents.

**Methods:**

Male adolescents (n = 5; 14–15 years old) from Yogyakarta wore accelerometers and automated wearable cameras for four days, and completed diaries and interviews in November 2020.

**Results:**

Participants’ activity was dominated by light intensity (67% of all physical activity). Sedentary behaviour was high; accelerometer, school days: 456 ± 145 min (78 ± 10% of wear time), non-school days: 344 ± 160 min (79 ± 17% of wear time); camera, school days: 176 ± 101 min (81 ± 46% of wear time), non-school days: 210 ± 165 min (86 ± 67% of wear time). Sedentary behaviour was mainly done during school hours on school days and from late afternoon to evening on non-school days. Screen time was largely for leisure purposes and action games were most favoured. Smartphones were the most used device, mainly used in a solitary context in the bedroom. Non-screen-based sedentary behaviour was consistently low. Interviews suggested that during the pandemic, supporting factors for physical activity are: self-determination, enjoyment, parental support, and physical education; meanwhile, factors influencing screen time are: educational demands, device and internet availability, screen time opportunities, parental control, social facilitators, phone notifications, and emotional state.

**Conclusions:**

Most participants were not able to stay active during the pandemic. Using digital platforms may be beneficial to shift some screen-based sedentary behaviour to ‘screen-based’ or ‘screen-prompted’ physical activity.

**Supplementary Information:**

The online version contains supplementary material available at 10.1186/s44167-022-00014-0.

## Background

The emergence of the COVID-19 pandemic has changed human behaviour significantly. The first case of severe acute respiratory syndrome coronavirus 2 (SARS-CoV-2) (COVID-19) was identified in Wuhan City, China in December 2019 [1]. The disease rapidly spread across the world and in March 2020 it was classified as a pandemic by the World Health Organization [2]. To minimise the spread of the outbreak, countries implemented social restrictions and closed public venues, including schools and sports facilities [3, 4]. Studies showed that socio-behavioural restrictions during the pandemic have caused a significant decrease in physical activity [5, 6] and an increased engagement in sedentary behaviour [5] among children and adolescents. These behaviours may cause serious health problems if persisting in the long term.

Previous studies provided important knowledge of how the pandemic affected physical activity and sedentary behaviour (for instance, see [7–11]) and some have tried to investigate the correlates of such behaviour change [12–14]. However, more information is needed to understand both behaviours more comprehensively. For example, apart from the duration and changes of physical activity and sedentary behaviour during the pandemic, it is also important to know when both behaviours are mostly done (the patterns), where (the physical setting), with whom (social interaction), and why (the reasons for behaviours). Knowledge of these behaviours may help stakeholders and researchers to develop policies and design interventions to handle future similar crises.

Furthermore, previous studies during the pandemic have heavily relied on online survey measurements [7, 10, 15], with known limitations including recall bias [16]. It is important to employ several instruments to understand physical activity and sedentary behaviour during the pandemic more comprehensively. The use of accelerometers is recommended to assess the duration, intensity and patterns of sedentary and movement behaviours [16]. Automated wearable cameras have become available to capture types, locational, and social contexts of behaviours [16]. Despite the strengths, wearable cameras are still rarely used in physical activity and sedentary behaviour studies [16–18]. Furthermore, the use of self-report diaries is needed to report data that devices would have missed when participants had to remove their devices, for example, when in a changing room or toilet. Lastly, by using interviews, the reasons for activities can be explored. The combination of these four methods to investigate both physical activity and sedentary behaviour (screen- and non-screen based) provides strength and novelty to the present study. To the best of our knowledge, none of previous studies during the pandemic used the combination of accelerometers, automated wearable cameras, diaries, and interviews (see Stockwell et al. [5]).

Research shows that adolescents have a high prevalence of insufficient physical activity (81%) [19] and have become the most sedentary group across the pediatric population [16]. Hence, more research on adolescents is needed, especially as a recent review revealed that less than 10% of studies examining physical activity and sedentary behaviour during the pandemic investigated the adolescent population [5]. The present study focused on male adolescents as this group has been identified as more active than their female counterparts [19, 20] but has had a greater decrease in physical activity during the pandemic [6]. Compared to girls, boys have also been found to have different patterns of sedentary behaviour and have different interests in screen time [21, 22]. However, little is known about the contexts or reasons for both behaviours during the pandemic solely in male adolescents [5, 12, 23]. Profound and comprehensive investigations on both behaviours also need to be conducted more in low- and middle-income countries (LMICs) [20, 24], including Indonesia [25].

Due to the social restrictions during the pandemic, as well as the nature of our data collection, which is intensive, somewhat burdensome and intrusive, we limited recruitment to five male Indonesian adolescents. Our study is a mixed-method case study that focuses on in-depth and integrative data collection and analyses of physical activity and sedentary behaviour. We argue that investigating such a sample in this way is as important as investigating a large sample but with less depth and diversity of assessment. Specifically, our case study aimed to investigate physical activity and sedentary behaviour in a small group of male Indonesian adolescents during the pandemic by examining behavioural quantity, temporal patterns, contexts, and underlying biopsychosocial factors, using mixed methods.

## Methods

### Study design

The design of this study was a case study [26] and employed the convergence model of a mixed-method approach, where quantitative and qualitative data were collected, followed by analysing both types of data simultaneously to derive a more comprehensive understanding of the findings [27]. The data collection site was in Yogyakarta, Indonesia. We collected data during the COVID-19 pandemic in November 2020, when participants received online education from home as schools were closed. Research packs and devices were delivered to participants and returned by following COVID-19 health and safety protocols.

### Participants

We recruited five male adolescents (age 14–15 years old) in an urban/suburban area of Yogyakarta Province, Indonesia. The recruitment of participants was part of a larger study before the COVID-19 pandemic. We called prospective participants to seek their interest to participate in the study. We delivered the research information to the participants’ home addresses and called the participants’ parents to explain the study. We encouraged participants to follow the study protocol by contacting them through the WhatsApp platform. Upon completion of the data collection, participants were offered a grocery voucher valued at ~ AUD ($) 20 as a token of appreciation for their time.

### Measures

#### Sociodemographic questionnaire

Participants and their parents were asked to complete a brief questionnaire relating to demographic characteristics, such as age, educational background, occupation, and availability of screen-based devices at home.

#### Anthropometry

We measured height to the nearest 0.1 cm by using a wall-mounted stadiometer and measured body weight (light clothing but no shoes) to the nearest 0.1 kg by using a digital scale. We calculated body mass index (BMI) by using BMI Percentile Calculator for Child and Teen [28].

#### Accelerometer

We used the ActiGraph wGT3X-BT accelerometers (ActiGraph, LLC, Florida; hereafter ‘ActiGraph’) to assess the duration and temporal pattern of physical activity and sedentary behaviour. Details of the accelerometers had been described in previous literature [17]. Participants wore the accelerometer during waking hours for 4 days (three online school days, and one non-school day) as the accelerometer measurement of 4 days was comparable to 1 week [29].

We followed the protocol to wear and process ActiGraph by Chandler et al. as they found a favourable accuracy to classify activity intensities [30]. Participants wore the ActiGraph on the non-dominant wrist to minimise “noise” during certain sedentary behaviours [31]. The results between waist- and wrist-worn accelerometers were comparable [32] and children reported higher compliance for wrist-worn compared to hip-worn accelerometers [32, 33]. Participants should remove the device during water activities, such as taking a shower and swimming.

Consistent with previous studies in adolescents, we set a sampling frequency of 30 Hz [34]. The time of the ActiGraphs was synchronized with the time of the wearable cameras (see later). We set the accelerometer filter to normal as the low-frequency filter will result in a decrease in the amount of sedentary time and greater time in physical activity [34].

#### Accelerometer data processing

In line with previous literature, we utilized Vector Magnitude, which is the square root of the sum of squared activity counts from three axes, to process accelerometer data [34]. Data were processed by using ActiLife Software (v6.13.4). We defined 20 min of consecutive zeros of the ActiGraph count per minute as non-wear time [34] and referred to Chandler et al. [30] for classifying activity cut points (see Additional file 1: Table S1). We conducted data cleaning and processing in May 2022.

#### Automated wearable camera

We used Brinno TLC120 automated wearable cameras (Brinno Inc, Taiwan) to assess types and contexts of physical activity and sedentary behaviour, including main behaviour, posture, device, content, activity, purpose, physical setting, and social contexts. Details of the camera had been described in previous literature [17]. The camera automatically captured an image every ~ 10 s then converted the images to a time­lapse video (.avi) and stored it on an SD card. A new video is created every time the on/off button is pressed. We converted the videos into single images (.jpg) manually by using the open-source software FFmpeg (version 4.3).

We adhered to the ethical framework for human research using wearable cameras by Kelly et al. [35]. Participants wore the camera on an adjustable chest-mounted harness. Simultaneously with wearing the accelerometer, participants wore the camera during their free time on three online school days from 3 p.m. until just before bedtime, and during waking hours on one non-school day. Third parties could ask participants to turn off the camera and ask to delete their images by contacting the researcher. Participants got a reference card containing an explanation about the device and the researcher’s contact details should any third party enquire about the camera. For ethical reasons, our study participants removed their devices if they needed to protect privacy, such as when in a changing room or toilet.

Participants and one of their parents had an opportunity to review and delete any collected images prior to the lead author viewing and analysing the images. The researcher, for privacy reasons, did not provide a copy of the images under any circumstances. The remaining images after any deletions were securely stored in a password-protected device and on a password-protected storage server. Images could only be accessed by the lead author (FDA), who acted as the main image coder, and one other researcher (AAP) who acted as a second image coder. The second image coder accessed a subset of images (~ 10%), as set by the lead author, to check for coding agreement.

#### Camera data processing

**Fig. 1:**
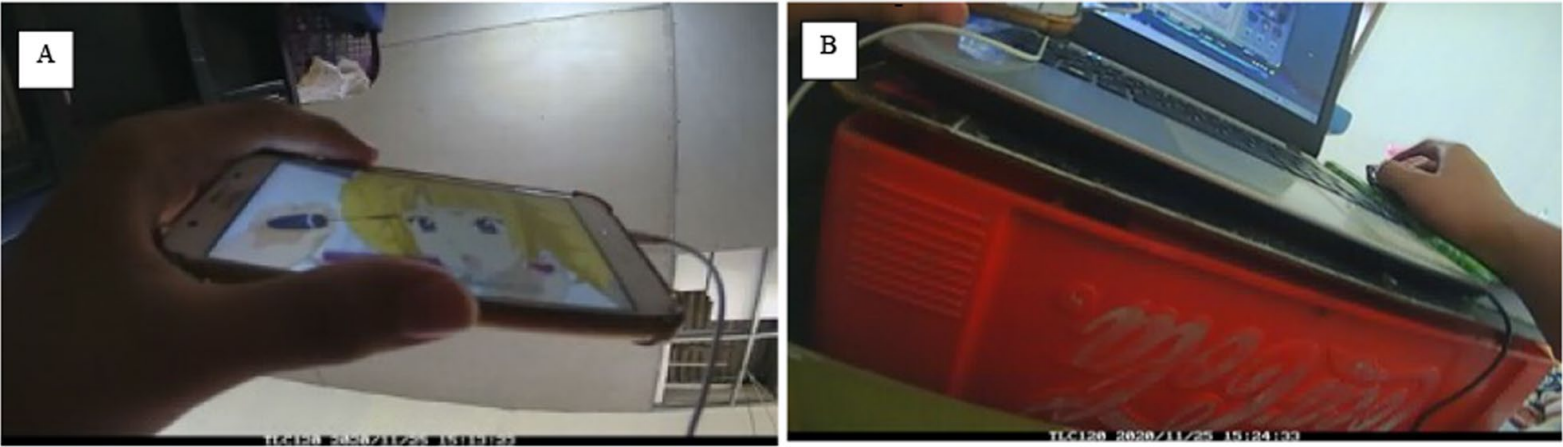
Sample of images and coding. **A** Date: 25/11/2020; Time: 15:13; Main behaviour: screen-based sedentary behaviour; Posture: Sitting; Device attention: primary; Device: Portable-mobile device (phone, ipod); Content type: passive screen media-animation/cartoon; Purpose: leisure; Physical setting: home: bedroom; Social context: alone; Social environment: alone; Social interaction: none; Other behaviour: none. **B** Date: 25/11/2020; Time: 15:24; Main behaviour: screen-based sedentary behaviour; Posture: Sitting; Device attention: primary; Device: Portable-laptop computer; Content type: interactive screen media-game-unclassifiable; Purpose: leisure; Physical setting: home: bedroom; Social context: alone; Social environment: alone; Social interaction: none; Other behaviour: none; Other Device: Portable-mobile device (phone, ipod); Device attention: secondary; Content type: passive screen media-animation/cartoon; Purpose: leisure

Image coding was completed between August 2021 and May 2022. We followed a guideline to code images that was used in a previous study [17] (see Additional file 2: Table S2). The lead author manually coded images by using Excel spreadsheets. Content types were classified into passive, interactive, and social media. Passive screen media involves activities where participants received screen-based content passively [36], such as watching TV programs. Interactive screen media involves cognitive or physical participation during screen time, such as typing on a laptop and playing video games [36]. Images that were blurry, blocked or in poor lighting were coded as such, or coded based on the preceding and subsequent images. Figure 1 shows examples of images and coding. We checked percent agreements [37] between the main and the second coder across ~ 10% of the images (n = 2907 images) and 8 coding categories (main behaviour, posture, device, content type, activity, purpose, physical setting, and social interaction). On average, we found 98.5% agreement.

#### Diary

Participants were provided with a pre-formatted diary (a small paper-and-pencil logbook) during data collection to record activities only whenever taking off the camera and/or the accelerometer, such as when taking a shower. The diary recorded the date, what device was removed, the time when removing the device (e.g., 15.00–15.30), and the activity when removing the device. It was also used to record bedtime and wake-up time.

#### Interview

The lead author interviewed each participant once through a phone call (mean duration: 51 min). The contexts and reasons for participants’ physical activity and sedentary behaviour were explored during semi-structured interviews. We utilised Olympus DS-3500 (Olympus Imaging Corp, China) and Olympus ME33 (Olympus Imaging Corp, Taiwan) to record the interviews. The interview guideline can be seen in Additional file 3.

#### Data analysis

Descriptive analysis was performed by using Microsoft Excel for Microsoft 365 MSO (16.0.14228.20200). We incorporated diary and accelerometer data to capture any missing activities and to cross-check non-wear time in accelerometer data. Activities that were mentioned in the diary were converted into relevant intensity by following the Youth Compendium of Physical Activities [38]. For the combined accelerometer and diary data, we provided descriptive data for wear time and duration of behaviours on both the online school days and non-school day. We presented the combined accelerometer and diary data into different time segments to analyse temporal patterning; on online school days: before school (before 07.00 h), during school (07.00 h to 15.00 h), late afternoon (15.00 h to 18.00 h), and evening (18.00 h to sleep); on the non-school day: morning (before 12.00 h), early afternoon (12.00 h to 15.00 h), late afternoon (15.00 h to 18.00 h), and evening (18.00 h to sleep).

For camera data, descriptive data were provided to show the total number of images, camera wear time, duration and number of images for each behaviour on the online school day and non-school day. We defined camera wear time as the total number of minutes the camera was turned on. Captured time (in minutes) was defined as the number of images divided by 6 (assuming each image represents 10 s). We split camera data to analyse temporal patterning; on the online school day: late afternoon (15.00 h to 18.00 h) and evening (18.00 h to sleep); on the non-school day: morning (before 12.00 h), early afternoon (12.00 h to 15.00 h), late afternoon (15.00 h to 18.00 h), and evening (18.00 h to sleep).

Interview recordings were transcribed verbatim and anonymised. We used NVivo software (Version 12 Pro, QSR International, VIC, Australia) to facilitate interview analysis. We adapted the reflexive thematic analysis approach to work with interview data [39, 40] with the steps as follows: (1) Data familiarisation, (2) Coding, (3) Generating initial coding groups, (4) Reviewing coding groups, (5) Integrating coding groups with results from accelerometers, wearable cameras, and diaries. The lead author (FDA), who interviewed all participants, became familiarised with the dataset by reading the transcripts. Subsequently, she coded the transcripts, followed by placing related codes into initial coding groups. Afterwards, initial coding groups were reviewed and revised. Lastly, coding groups and codes were integrated and triangulated with results from device-based measurements and diaries. Interview data were analysed between June and July 2022.

## Results

We present results from accelerometers, diaries, automated wearable cameras, and interviews both separately and combined to enable better clarity, integration and triangulation of findings.

### Sample characteristics

The characteristics of the participants are presented in Table 1.

**Table 1:** Characteristics of participants (*n* = 5)

Variable	PM1	PM2	PM3	PM4	PM5
Age (years)	14.6	14.6	14.3	14.6	14.9
Height (cm)	170.2	168.6	153	165	173
Weight (kg)	49.7	68.2	37.6	63.6	63.8
Body mass index	17.2	24.0	16.1	23.4	21.3
Mothers’ age (years)	44.6	45.3	41.8	43.9	53.3
Number of people in the household	5	7	6	5	4
Mothers' highest level of education					
Primary school					√
Year 12 or equivalent	√	√	√	√	
Mothers’ occupation					
Entrepreneur		√	√		
Homemaker	√			√	
Cleaner					√
Screen-based devices at home					
TV	√	√	√	√	√
Laptop			√	√	
Smartphone/iPhone	√	√	√	√	√
Video games connected to TV (e.g., Playstation/Xbox)	√		√		
The most used social media account					
Whatsapp	√	√	√	√	√
Instagram	√	√	√	√	√
Parent’s social media account					
Facebook			√		√
WhatsApp	√	√	√	√	√

### Wear time

The accelerometer wear time combined with diary data (see "Data analysis") during a school day was higher than that for a non-school day, range 260–723 min vs 91–735 min. Meanwhile, the range of camera wear time on a school day and a non-school day was 100–368 min and 43–465 min, respectively. A total of 26,979 images, derived from 15 school days and 5 non-school days, were coded and analysed. On average, the camera captured 1308 ± 664 images on a school day and 1471 ± 1090 images on a non-school day.

### Duration of physical activity and sedentary behaviour

Table 2 shows a summary of the accelerometer data combined with diary data. Participants spent a high proportion of their time in sedentary behaviours (range: 210–577 min or 62–89% of wear time) on a school day and (range: 69–465 min or 52–95% of wear time) on a non-school day. Excluding one participant (PM5), Participants’ physical activity was dominated by light intensity, both on a school day and a non-school day, range: 47–162 min and 20–79 min, respectively.

**Table 2:** Summary of time spent in physical activity and sedentary behaviour based on accelerometer data combined with diary data

Participant code (n = 5)	SB		LPA		MPA		VPA		Accelerometer wear Time + diary data
	Minutes	%	Minutes	%	Minutes	%	Minutes	%	Mean (minutes)
Online school day (M)									
PM1	538	76	162	23	13	2	0	0	713
PM2	577	83	114	16	4	1	0	0	695
PM3	210	81	47	18	3	1	0	0	260
PM4	504	89	63	11	1	0	0	0	569
PM5	450	62	105	15	163	22	5	1	723
Total mean	456	78	98	17	37	5	1	0	592
SD	145	10	45	4	71	9	2	0	196
Weekend day									
PM1	363	88	48	12	1	0	0	0	412
PM2	465	85	79	14	4	1	0	0	548
PM3	69	76	20	22	2	2	0	0	91
PM4	446	95	24	5	0	0	0	0	470
PM5	379	52	59	8	291	40	6	1	735
Total mean	344	79	46	12	60	9	1	0	451
SD	160	17	25	6	129	18	3	0	235

*SB* sedentary behaviour, *LPA* light physical activity, *MPA* moderate physical activity, *VPA *vigorous physical activity

The automated wearable cameras showed similar results where the majority of participants’ camera wear time contained screen images (weekday: range 65–89%; weekend day: range 62–97%). Please note that the most active participant (PM5) removed the devices during his sports training, but we were able to capture the duration of his training from diaries, which we integrated with accelerometer data. The proportion of non-screen-based sedentary behaviour was consistently low (weekday: range 3–26%; weekend day: range 0–24%). Details of camera data are presented in Table 3.

**Table 3:** Summary of time spent in physical activity and sedentary behaviour based on camera data^a^

	PM1		PM2		PM3		PM4		PM5		Total mean		SD	
	School (M)	Weekend	School (M)	Weekend	School (M)	Weekend	School (M)	Weekend	School (M)	Weekend	School (M)	Weekend	School (M)	Weekend
Number of images	1463	256	2207	1643	703	477	1568	2793	602	2184	1308	1471	664	1090
Time of the first image, h:min	15:55	14:52	15:14	10:02	15:01	17:19	15:13	13:46	15:19	6:39	15:20	12:31	0:20	4:12
Time of the last image, h:min	20:38	15:35	22:54	19:51	19:18	19:58	19:56	21:58	18:52	14:01	20:19	18:16	1:35	3:19
Wear time, h:min^b^	4:03	0:43	6:08	4:35	1:56	1:19	4:21	7:45	1:40	6:05	3:38	4:05	1:50	3:01
Captured time, min^c^	244	43	368	274	116	79	261	465	100	364	218	245	111	182
Physical activity images, number	102	15	144	25	51	32	32	28	53	276	76	75	46	112
Physical activity, min^c^	17	3	24	5	8	6	5	5	9	46	13	13	7	18
Screen-based PA images, number	24	5	6	0	1	0	1	7	1	29	6	8	10	12
Screen-based PA time, min^c^	4	1	1	0	0	0	0	1	0	5	1	1	2	2
Screen-based SB images, number	941	236	1915	1559	560	445	1399	2709	460	1354	1055	1261	606	989
Screen time, min^c^	157	39	319	259	93	73	233	451	77	226	176	210	101	165
Non-screen-based SB images, number	386	0	141	59	21	0	65	49	55	525	134	127	148	224
Non-screen-based SB time, min^c^	64	0	24	10	4	0	11	8	9	87	22	21	25	37
Uncodeable images, number	11	0	1	0	70	0	71	0	33	0	37	0	33	0

^a^Included 20 days (15 school day evenings and 5 non-school days) from five participants.

^b^Minutes the camera was turned on.

^c^One image represents 10 s (number of images/6).

h: hour; min: minute; PA: physical activity; SB: sedentary behaviour; M: Mean of three days; SD: standard deviation; school: online school day

Interviews revealed the contexts of these results. Participants reported doing more physical activity before compared to during the pandemic. Before the pandemic, participants joined structured physical activity programs, either at school (sports extracurricular) or in the community (sports club). They reported often doing physical activity after school, such as playing soccer and volleyball. Interviews suggested that the most supporting factors for participants’ physical activity before the pandemic was the availability of friends and open spaces, as well as parental support.

“Before the pandemic, school finished at 2:30 (pm). Usually (I) play soccer at school and go back home around 4:30 (pm). I did it almost every (school) day. I didn’t play (soccer) and directly go home after school only when there are no friends to play with” (PM1).

“Mm.. (before pandemic) after school (I) play soccer one to two hours in a field (near the house)” (PM2).

Note: PM = Participant

Barriers for physical activity during the pandemic were revealed. Firstly, participants mentioned that the absence of friends demotivated them from being active. They mentioned that their friends were not willing to play physical games during the pandemic, even though it was allowed, as they were more into screen-based games. This has made these participants tend to withdraw from physical activity and chose to do more screen-based activities.

“For physical activity, my friends are not.. not willing to do it anymore. Friends who live nearby don‘t willingly go running or that kind of thing. If.. (doing physical activity) by myself, I am not sure to do that” (PM1).

“I still can do physical activity, but very rarely”; (Interviewer: Why?) Mm.. (I) rarely go out, usually, my friends don’t want to do that (physical activity) (PM4).

**Fig. 2:**
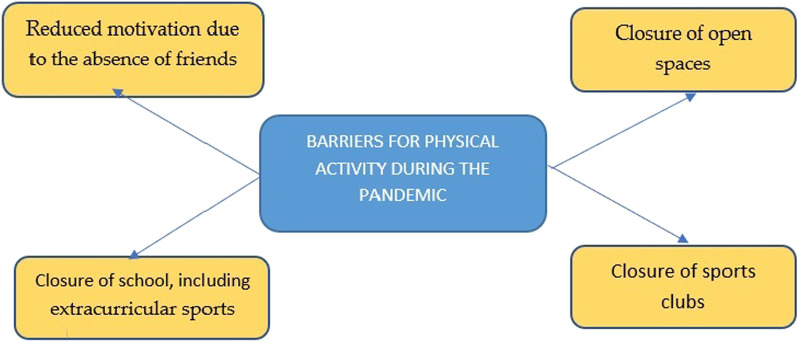
Barriers for doing physical activity among male adolescents during the pandemic

Moreover, the closure of open spaces near participants’ homes during the pandemic also hampered them in doing group physical activity. Participants also could not join sports clubs and extracurricular sports given that they were closed. Barriers for doing physical activity during the pandemic can be seen in Fig. 2.

**Fig. 3:**
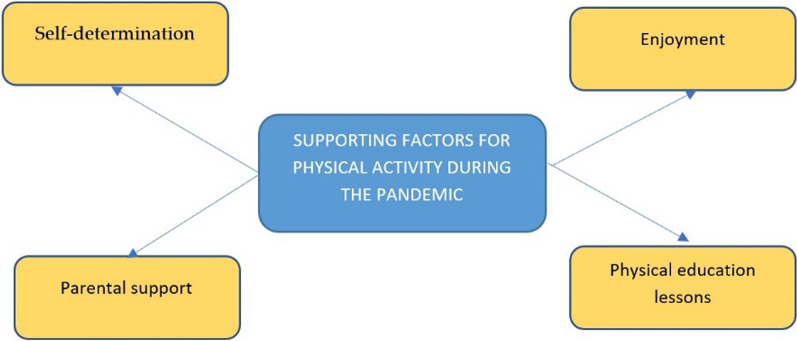
Supporting factors for physical activity among male adolescents during the pandemic

Accelerometer data showed only one participant (PM5) allocated a relatively significant proportion of his time for physical activity during the pandemic (weekday: 273 min, weekend day: 356 min). The interview showed that this participant joined a sports club in the community before the pandemic and that he keep doing physical activity regularly during the pandemic, such as jogging around his house. These statements are confirmed by automated wearable camera data. Being supported by his parents, some months after the pandemic began, he registered and moved to another volleyball club that offered training during the pandemic. The high level of self-determination, enjoyment, and parental support (e.g., support to join the club and provide transport), seemed to be the most supporting factors for doing physical activity in a difficult situation during the pandemic. Participants mentioned physical education lessons facilitated them to do some forms of physical activity through school assignments. Supporting factors for physical activity during the pandemic can be seen in Fig. 3.

“During pandemic.. there are assignments (from physical education teacher) such as gymnastics, basketball, volleyball, disc throw” (PM3).

Interviews revealed that participants did much less screen time before the pandemic as they cannot use their screen-based devices during school hours. Participation in structured physical activity and other physical activity forms after school also kept them away from leisure screen time. This was not the case during the pandemic when they engaged in screen time more frequently.

**Fig. 4:**
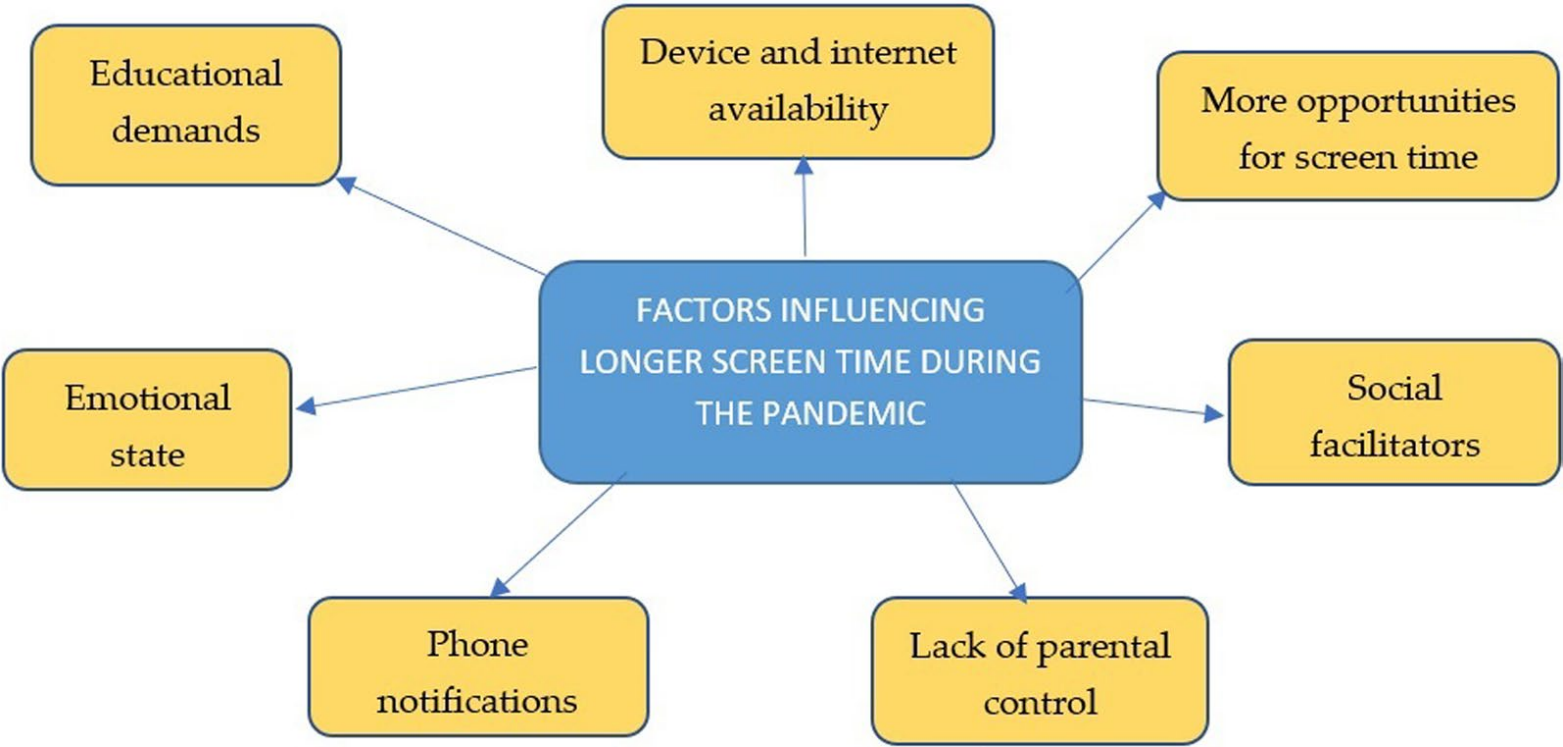
Factors influencing screen time among male adolescents during the pandemic

Factors that appeared to influence longer screen time during the pandemic are presented below and in Fig. 4. Firstly, the implementation of online schooling made participants rely on screen-based devices for studying and working on assignments. Secondly, access to screen-based devices, such as smartphones, and the internet, facilitated participants for doing more screen time. A participant said that his parents bought him a laptop computer at the beginning of the pandemic to facilitate online study, but camera data showed that he also used the device a lot for playing online games. Thirdly, a higher opportunity to engage with screen-based devices. Participants mentioned how they were able to do recreational screen time in the middle of doing school assignments and that they tend to sleep late at night which increased the chances for screen time. The availability of friends or other family members to play screen-based games, such as PlayStation and online games, also increased participants’ screen time considerably. Most participants mentioned how their friends often invited them to play online games together. Furthermore, inconsistency or unavailability of screen time rules from parents seemed to cause screen time to be longer.

“During the pandemic, my Mom is tired of reminding me (to reduce screen time), because (I) always use my phone” (PM1).

“(My parents) often gave me advice (to limit screen time), but (laughing) (I) rarely obey that” (PM4).

Phone notifications also influenced participants’ screen time as it triggers them to check their phones more frequently. However, the use of notifications appeared important during online school as participants needed to keep up to date with school-related information which could only be accessed using screen-based devices.

“Mm.. if there is an assignment (notification) from school, I checked (my phone). There was once (my phone) on a silent mode, then I missed a lesson” (PM4).

Lastly, the emotional state also appeared to influence screen time. Participants mentioned feeling bored as a reason to escape to screen-based activities.

“Yes, when I feel bored, then (I) watched YouTube, (checked) social media, and played games” (PM2).

“Yes, because of feeling bored, how to say? Mm.. because I feel bored so I can’t, sometimes I can’t get off (from phone). Usually if there is no one asking me to go out I can use the phone all day ” (PM4).

### Temporal patterns of physical activity and sedentary behaviour

**Fig. 5:**
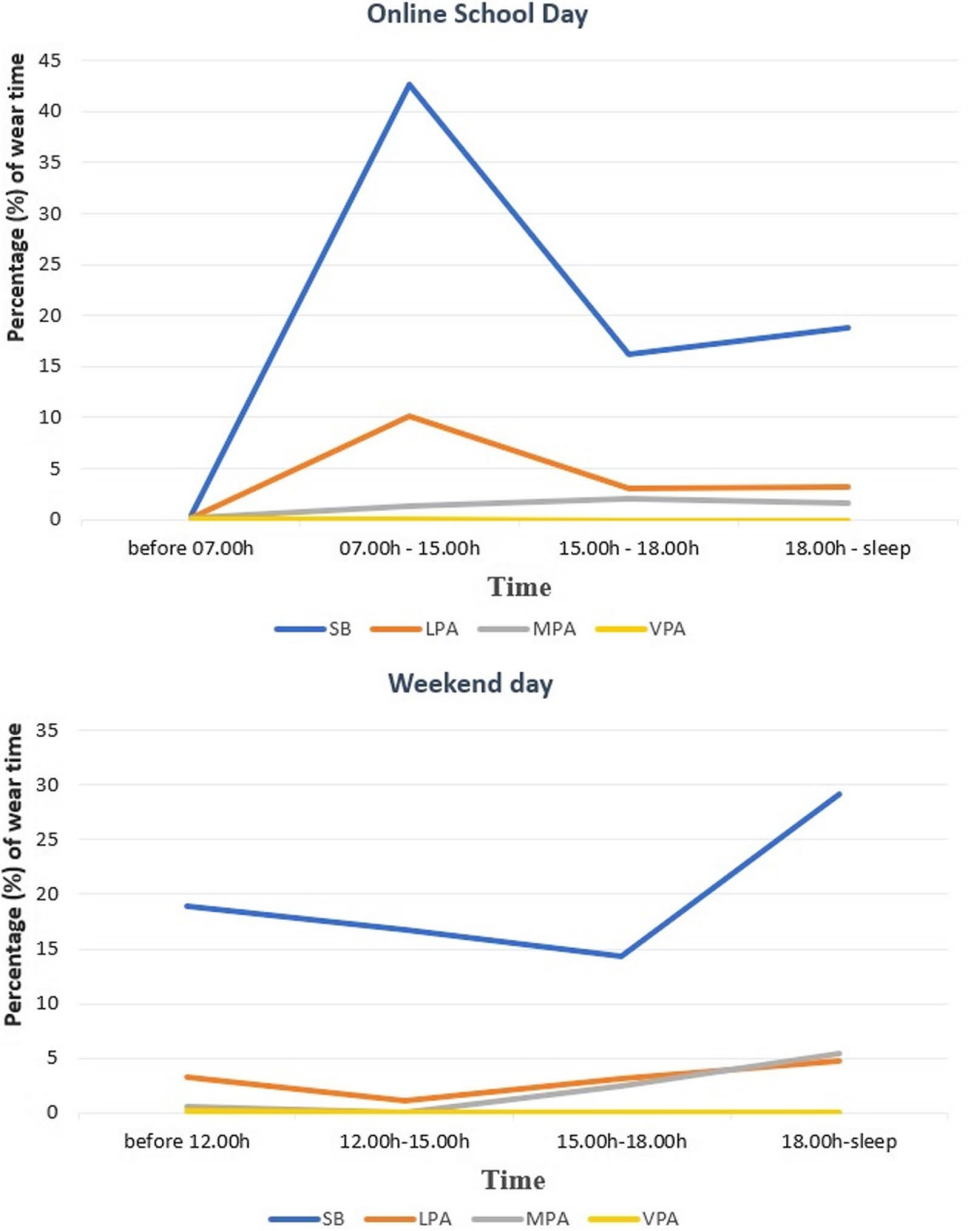
The temporal patterns of activity based on accelerometer and diary data. *SB* sedentary behaviour, *LPA* light physical activity, *MPA* moderate physical activity, *VPA* vigorous physical activity

Figure 5 illustrates the temporal pattern of participants’ activity for the school and non-school days based on accelerometers combined with diary data. On school days, participants were mainly sedentary during school hours, from 07.00 to 15.00 (43% of wear time). However, interviews showed that participants’ sedentary behaviour during this time frame was not only for educational purposes. Participants mentioned they could finish assignments in a couple of hours or do that later in the day and stated doing leisure screen time during school hours; but we cannot capture the content as, for ethical reasons, participants wore the automated camera after school hours. The pattern for physical activity of any intensity was low throughout the day (≤ 10% of wear time on each time point). The pattern, however, was much different on the non-school day where participants did sedentary behaviour much more in the evening (29% of wear time). Physical activity of any intensity remained low during the non-school day (≤ 5% of wear time on each time point).

**Fig. 6:**
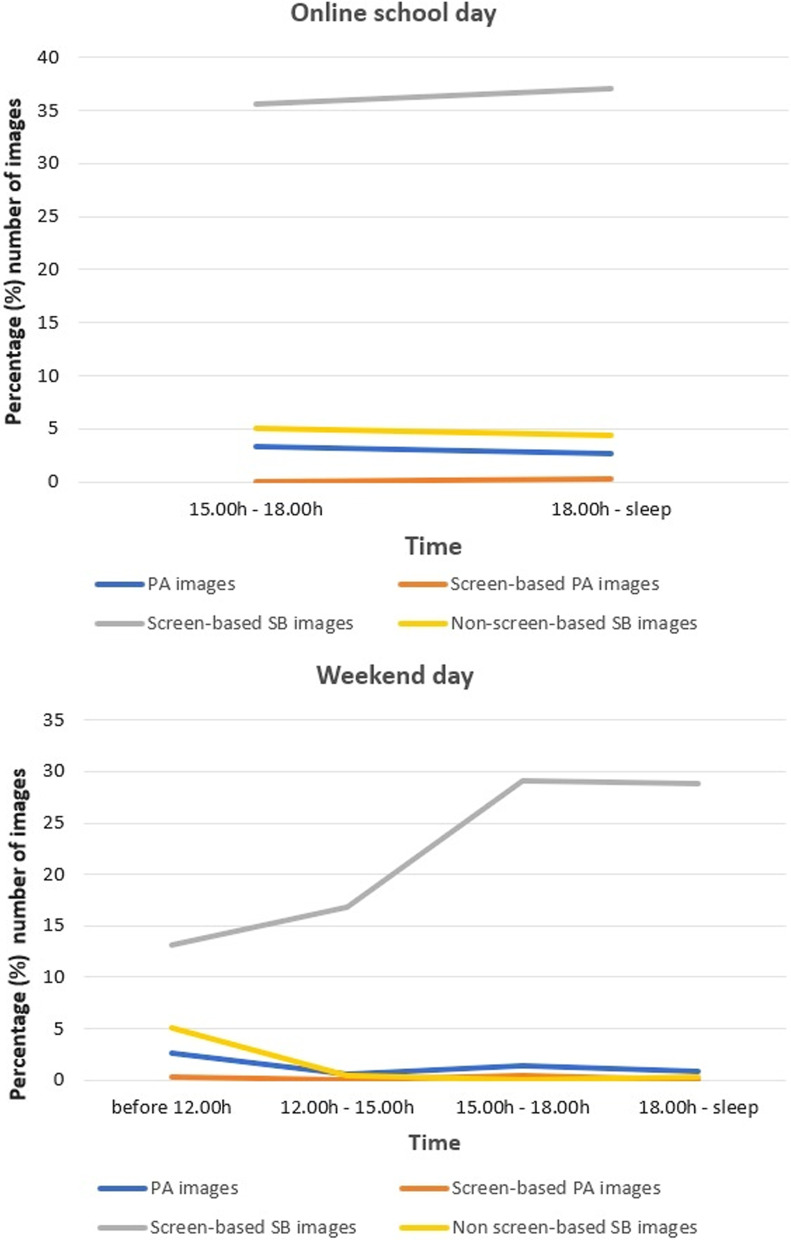
The temporal pattern of activity based on camera data. *PA* physical activity, *SB* sedentary behaviour

Figure 6 shows the patterns of participants’ activity based on the number of camera images for both a school- and a non-school day. During school days, participants’ activity in the late afternoon and evening was mainly screen time (36% and 37% of wear time, respectively). Screen time was also dominating participants’ activity during the non-school day, with the peak from late afternoon to evening (29% of wear time each). Non-screen-based sedentary behaviour and physical activity were consistently low both on a school- and a non-school day.

### Types, contents, and purposes of physical activity and sedentary behaviour

Camera data showed that movement behaviours comprised the least proportion of participants’ behaviour (6% of total images). Of this, the most frequent activity was walking inside the house, primarily to move from one room to another (30% of movement behaviours).

Screen time accounted for the biggest percentage of other participants’ behaviour (82% of total images).

The most used device was the smartphone (74% of screen time). Of this, interactive screen media dominated the content of participants’ screen time, particularly action games (29% of phone use). *Mobile Legend* was the most cited online game by participants in interviews. Passive screen media was also relatively popular, especially cartoon/animation programs (13% of phone use) and live-action programs (12% of phone use). The most popular social media was WhatsApp (9% of phone use).

Seventeen per cent of participants’ screen time was for watching television. Of these, the live-action program was the most popular (88% of television-watching). Television for gaming (PlayStation) was accessed only by one participant (PM3) who used that mainly for sports games (97% of television for gaming).

Both camera data and interviews showed that participants preferred to use their smartphones over television for watching cartoons and live-action programs, such as online games, sports, and hobby-related content on YouTube. These are mostly available at any time on YouTube, unlike some television, and more accessible as participants can watch them by using their phone in their room without the need to go to the television room.

Regarding social media, in line with camera data, interviews revealed that WhatsApp was the most accessed app and it was mainly used for school-related purposes as most school assignments and information were informed through this platform.

“(I) often check WhatsApp, school groups, that’s for.. like.. checking information about recent assignments, checking information from school groups” (PM3).

Another app mentioned, but less accessed by participants was Instagram. Participants said that they were not keen on posting on this social media platform, and used the app mostly for checking updates from others and looking for information, especially related to their hobbies.

“For Instagram, (I used it) only for seeing other’s posts, then also for looking game information, and soccer clubs” (PM1).

Non-screen-based sedentary behaviour comprised 10% of total images. Of this, the highest percentage was for sitting/lying/reclining (32%) and socializing (27%). Writing/doing school work and reading educational books accounted for 10% and 3% of non-screen-based sedentary behaviour, respectively. Details of types and content of behaviours can be seen in Table 4.

**Table 4:** Type and content of behaviours based on camera data

Variable	*n* of images	%
Screen-based sedentary behaviour	22,131	82
Non-screen-based sedentary behaviour	2637	10
Movement behaviours	1655	6
Uncodeable	557	2
Sum	26,980	100
Types of movement behaviours		
Physical activity: walking	503	30
Unclassifiable	422	25
Physical activity: conditioning exercise	248	15
Screen-based physical activity	132	8
Other	111	7
Chore (e.g., cleaning the house, washing dishes)	83	5
Physical activity: sports: ball sport	60	4
Socializing (with some movement)	62	4
Reading: other (with some movement)	12	1
Reading: unclassifiable (with some movement)	9	1
Drinking: beverage (with some movement)	7	0
Self-care (e.g., taking a shower, brushing teeth)	6	0
Sum	1655	100
Types of screen-based sedentary behaviour		
Content		
Portable device: mobile phone/smartphone		
Interactive screen media: game: action	4770	29
Unclassifiable	3168	19
Passive screen media: programme: animation/cartoon	2063	13
Passive screen media: programme: live action	1922	12
Social media: Whatsapp	1508	9
Interactive screen media: other	639	4
Passive screen media: unclassifiable (e.g., looking at something)	627	4
Passive screen media: programme: live action animation	601	4
Social media: instagram	478	3
Interactive screen media: internet: browse (e.g., online shopping)	163	1
Passive screen media: internet: article, book, blog	151	1
Interactive screen media: unclassifiable (e.g., scrolling, browsing)	137	1
Passive screen media: programme: unclassifiable	94	1
Interactive screen media: game: unclassifiable	71	0
Interactive screen media: communication > online meeting	32	0
Passive screen media: general (e.g., home page, calculators)	21	0
Social media: other	16	0
Interactive screen media: communication > Call	10	0
Interactive screen media: creation: camera App (e.g., photo/video)	7	0
Sum	16,478	100
Portable device: laptop computer		
Interactive screen media: game: action	797	56
Unclassifiable	260	18
Interactive screen media: game: unclassifiable	244	17
Interactive screen media: unclassifiable (e.g., scrolling, browsing)	100	7
Passive screen media: general (e.g., home page, calculators)	12	1
Interactive screen media: internet: browse (e.g., online shopping)	2	0
Sum	1415	100
Non-portable device: television		
Passive screen media: programme: live action	3275	88
Passive screen media: programme: unclassifiable	423	11
Passive screen media: programme: live action animation	22	1
Passive screen media: programme: animation/cartoon	12	0
SUM	3732	100
Non-portable device: television for gaming		
Interactive screen media: game: sports	491	97
Interactive screen media: game: unclassifiable	15	3
Sum	506	100
Types of non-screen-based sedentary behaviour		
Sitting/lying/reclining	837	32
Socializing	701	27
Writing: doing schoolwork	267	10
Eating: meal	260	10
Eating: snack	182	7
Other	173	7
Reading: educational book	83	3
Unclassifiable	62	2
Drinking: beverage	36	1
Reading: other	14	1
Reading: unclassifiable	14	1
Sum	2637	100

We were not able to identify the purpose of more than half of the movement behaviour images. Eighteen per cent of the images were for personal care (18%), such as conditioning exercises for health-related purposes. Meanwhile, screen time was done mainly for leisure purposes (63% of smartphone use; 74% of laptop use; 100% of television watching; 100% of television for gaming). The purpose of more than a quarter of non-screen-based sedentary behaviours was unclassifiable. Social and personal care, such as socializing and eating, were key non-screen-based sedentary behaviours (27% and 18%, respectively). Details of the purposes of behaviours can be seen in Additional file 4: Table S4.1.

### Physical setting

Camera data showed that sedentary behaviour was primarily done in or from a bedroom (smartphone: 88%, laptop computer: 100%, TV: 95%, television for gaming: 100%, non-screen-based sedentary behaviour: 32%). Some portions of watching TV were done in a room that looks like a bedroom but we cannot specify whose bedroom it was.

Physical activity was mostly done in and around the house (77%). However, we cannot classify the room for just over a quarter of physical activity images as they mainly showed participants moving or walking from one room to another. Details of the physical setting of activity based on camera data can be seen in Additional file 4: Table S4.2.

### Social context, environment, and interaction

Additional file 4: Table S4.3 illustrates the social context, environment, and interaction of participants’ activity based on camera data. The social context and environment for participants’ physical activity were mainly solitary (62%) and without social interaction (78%). Similar findings were found for screen-based sedentary behaviour, especially smartphone and laptop computer use, but not for television watching and television for gaming. Most smartphone and laptop computer use involved no social interaction (88% and 100%, respectively). Participants did much more social interaction when watching television (co-viewing: 45%) and using television for gaming (co-participating: 97%). While over half of non-screen-based sedentary behaviour was done alone, 43% of this behaviour involved direct social engagement.

## Discussion

Our study aimed to increase understanding of physical activity and sedentary behaviour in a small group of male Indonesian adolescents during the COVID-19 pandemic. Specifically, by adopting an intensive multi-method approach, we were able to study behavioural quantity, temporal patterns, contexts, and underlying biopsychosocial factors. This study, to our knowledge, is the first to use the combination of accelerometers, automated wearable cameras, diaries, and interviews to investigate physical activity and sedentary behaviour during the pandemic. We do not offer generalizability as it requires a larger sample, but instead, we provide a deep and comprehensive investigation from multiple points of view and assessments.

It is important to note that in Indonesia the first COVID-19 case was confirmed on March 2, 2020 [41]. We collected data in November 2020 in the Yogyakarta region when emergency response status and the closure of schools were still implemented to minimise the spread of the virus [42, 43]. Students had been learning online from home since the end of March 2020 [42].

In line with previous studies on Canadian children and youth [44, 45], our data from accelerometry combined with diary and camera data showed that during the pandemic most participants had low physical activity levels and that all participants allocated a significant proportion of their time to sedentary behaviour, especially using screens. Supporting findings from a recent systematic review involving adolescents in Asia, Australia, Europe, and America [5], our interview data suggested that compared to pre-pandemic, participants spent less time on physical activity and more time on screen-based sedentary behaviour. It seems clear that the COVID-19 pandemic has negatively affected physical activity and increased the sedentary behaviour of young people across the world, from LMICs to high-income countries.

Concerning the temporal pattern of physical activity and sedentary behaviour, to date, we did not find any previous study that investigate this during the pandemic. A previous study on Irish adolescents before the pandemic found that, on weekdays, boys were significantly more active just before and after the typical school day (8–10 am and 4–5 pm) than during school time (10 am–4 pm); while on weekends boys were significantly less active in the morning period (8–11 am) [46]. In contrast, our overall data revealed that the pattern of moderate and vigorous physical activity during the pandemic was low throughout the day, both on a school- and non-school day. In line with a previous study on female Indonesian adolescents before the pandemic [17], we found that the most prevalent physical activity intensity was light, which was most evident during the school day during school hours (7 am–3 pm). Our camera data provided information on the activity type, revealing that the most frequent activity across the day was walking inside the house, primarily moving from one room to another. It seems promising to increase the overall physical activity level by increasing the amount of light-intensity physical activity. Poitras et al. found that any physical activity, even though sporadic, provided benefits [47]. The benefits of light-intensity physical activity are now evident, including its positive contribution toward cardiometabolic biomarkers [47] and the decrease in depressive symptoms [48]. It may also provide a gateway to higher levels of activity intensity.

For physical activity, while our interview data showed that participants seemed to be active before the pandemic, our overall data showed that most participants appeared not able to keep their previous physical activity level during the pandemic. Chambonniere et al. [49] found a similar result with adolescents in France. In line with our findings, Yomoda et al. [6] showed that the decline in physical activity during the pandemic was more prevalent in boys and among children who participated in organized team sports. Dunton et al. suggest that changes in physical activity and sedentary behaviour during the COVID-19 pandemic may persist in the long term, which may increase the risk of obesity and cardiovascular disease in children [10]. Future studies should investigate the tracking of physical activity and sedentary behaviour after the pandemic.

In line with previous studies [12, 50], our interviews revealed that factors affecting participants’ physical activity during the pandemic included support from peers, family, and teachers; the availability of public open spaces near home, as well as the availability of structured physical activity, such as through sports clubs at the community. Similar to previous studies, we also found that a high level of self-determination [50], enjoyment [50–52] and parental support [50, 53] appeared to be the most supporting factors for maintaining physical activity. However, our interviews indicated the significant role of physical education (PE) to facilitate participants doing physical activity during the pandemic, especially when it is difficult to encourage participants to be physically active through other means. PE teachers may encourage and facilitate students to be physically active through various assignments, but preferably assignments that provide enjoyment and consider the students’ interests based on their characteristics to increase adherence. Free online resources were available to support PE teachers to design online PE during the pandemic (for example, see “50 Exercises and Activities for At-Home P.E.” [54] and “How to Stay Active at Home: PE At Home Resources” [55]).

Furthermore, our overall data showed that on school days sedentary behaviour was dominating participants’ behaviour throughout the day, with the highest percentage during school hours (07.00 h–15.00 h). However, as expected, our interviews revealed that participants’ screen time during school hours was not only for education but also for recreational purposes. We were not able to identify the screen time content during this time frame as participants wore the automated wearable camera after school hours only, due to research ethics constraints. Consistent with previous findings in female Indonesian adolescents and Australian adolescents before the pandemic [17, 18], our camera data showed that participants’ behaviour after school hours, from late afternoon to evening, was mainly screen time. This was also the case on the non-school day, but with the peak from late afternoon to evening. The temporal patterning of participants’ behaviour that we found may illustrate types of behaviours that attract these male adolescents’ attention at different periods of the day during the pandemic.

Regarding the context of the temporal patterning of behaviours, our interviews revealed similar findings as a previous qualitative study [50] showing that the high use of screen-based devices throughout the day in adolescents during the pandemic appeared to be associated with educational demands (learning online from home), devices and internet availability, lack of parental control, social facilitators from friends and family members (e.g., supports to play screen-based games), and psychological drawbacks due to the pandemic, which is often mentioned as “boredom”. Aligned with a previous qualitative study [22], we also found that phone notifications triggered participants to check their phones more frequently, but especially to check updates on school-related information.

The increase in adolescents’ screen time during the pandemic also appeared to be related to more opportunities for screen time throughout the day. This was not the case before the pandemic as adolescents may access screen-based devices primarily after school, thus this period of the day has been referred as the “critical hours” for physical activity and sedentary behaviour [56]. Adolescents’ minimal concern about how long they do screen time, and adolescents’ opinion that their usage is appropriate, might explain excessive screen time [57]. As suggested by previous literature, increasing awareness about the risks of excessive screen time should be a priority when developing strategies to modify screen-based sedentary behaviour in adolescents [58].

Due to deleterious effects that may be acquired from long periods of sedentary behaviour [59–61], efforts to reduce sedentary behaviour during the pandemic are warranted. Integrating physical activity into (non-PE) school lessons is not a new concept (see Parks et al. [62] and Reeves et al. [63]) and seems to be a promising method to reduce sedentary behaviour among students during school hours. The integration of physical activity into online lessons may provide benefits. Nevertheless, more needs to be done to convince teachers and education stakeholders about the importance of this issue. Teachers also need to have training to be able to incorporate physical activity effectively in their classes [62].

Similar to a previous study on adolescent girls in a LMIC before the pandemic [17], we found that the pattern of non-screen-based sedentary behaviour was consistently low on school- and non-school days. The highest percentage of non-screen-based sedentary behaviour was for social activities (e.g., having a conversation with others), personal care (e.g., eating), and educational activities (e.g., writing for school assignments). While screen-based sedentary behaviour was associated with adverse effects, in their systematic review, Carson et al. found that a longer duration of non-screen-based sedentary behaviour (i.e., reading and working on homework), was associated with more favourable academic outcomes [60]. Future studies may need to investigate the different effects of shifting screen-based sedentary behaviour to physical activity and non-screen-based sedentary behaviour, recognizing that some sedentary behaviours may be beneficial.

We found that the smartphone was the most used device for screen time, and this is in line with a previous study in Singapore [57]. This may be caused by multiple functions that are offered by the smartphone, from social activities through social media applications (e.g., WhatsApp), entertainment (e.g., through YouTube), functional purposes (e.g. browsing information on the internet), schoolwork (e.g., browsing information for assignments), to daily function (e.g., checking time), as well as the portability of the device [22, 57].

Furthermore, our camera data showed that there was a large gap between educational and leisure screen time during participants’ free time, with the majority of identified screen time being for leisure purposes. Our findings are consistent with a previous survey study by Dunton et al. [10] that found that children allocated around 90 min per day to educational-related sitting in their free time, but engaged in over 8 h for leisure-related sitting. Future studies need to examine the benefits and drawbacks of leisure screen time when combined with more ‘productive’ educational screen use [17].

In line with a previous study on female adolescents in a LMIC [17], we found that participants' sedentary behaviour was primarily conducted in the bedroom and that the smartphone was used mainly without social interaction. This may be due to greater privacy to access content with fewer interruptions from family members in this setting [18]. Our finding reflects the characteristics of adolescents who need to have more privacy and autonomy from their family [64, 65].

Regarding content, we found that interactive screen use, particularly action games, dominated participants’ screen time. This is consistent with a previous longitudinal study that found boys spend large amounts of time playing electronic games [21]. We also found that passive screen media was relatively popular, especially cartoon/animation programs and live-action programs. Sanders et al. found that the specific domain of screen use moderated educational and health effects [66]. For example, they found that educational screen time was associated with positive educational outcomes and higher persistence [66]. Meanwhile, interactive screen use (e.g. video games), had a positive correlation with educational outcomes but a negative correlation with health and socio-emotional outcomes [66]. Moreover, passive screen use was found to have a negative association with educational, health, and socio-emotional outcomes [66]. Therefore, facilitating behaviour change strategies for healthier leisure screen use is warranted for adolescents to minimize the negative impacts of screen time [50]. It is also important to provide suggestions on games that may facilitate physical activity and contribute positively toward psychological health and social interaction [67].

Given the high level of participants’ screen time during the pandemic, shifting the way participants use their screen-based devices from primarily screen-based sedentary behaviour to more ‘screen-based’ or ‘screen-prompted’ physical activity should also be considered. Digital platforms can support engagement in physical activity during movement restrictions, such as during the pandemic [68]. Examples of digital platform use include online streaming for exercise (e.g., via YouTube), subscribing to fitness programs, using an app to guide physical activity; participating in dance and fitness classes via platforms such as Zoom; using digital training or racing platforms (e.g., Zwift and Rouvy); and playing active electronic games [68]. Parker et al. found that the adherence of adolescents to physical activity guidelines was higher among users of these digital platforms compared to non-users [68].

The key strength of our study is that we used contemporary technology-based measurements, including accelerometers and automated wearable cameras, as well as diaries and interviews, while in the challenging situation of the COVID-19 pandemic. The instruments enabled us to explore a broad spectrum of physical activity and sedentary behaviour (both screen- and non-screen-based) and provided comprehensive and important information on male adolescent behaviours during the pandemic, including the duration, temporal patterns, content, and contexts. The combination of such methods enabled us to triangulate results from device-based measurements with diary and interview data so that we could elaborate and deepen our findings.

Despite the in-depth nature of the methods adopted, a limitation of our study is the small sample size. It is also important to consider that participants may have removed the devices when needed, such as removing the camera when doing sports or going outside their house, so our results may underestimate the actual duration of participants’ behaviours. While the use of diaries was intended to capture any behaviour when participants remove the devices, participants may have missed reporting some behaviours. Furthermore, while we asked participants to do their daily activities as normal during data collection, there is a possibility that participants changed some of their behaviour in reaction to the devices. We were also not able to identify the content of some parts of the camera images due to being out of frame.

## Conclusions

We found that during the pandemic our small sample of male adolescents allocated a substantial proportion of their time to sedentary behaviour. While before the pandemic participants seemed more active, most of them were not able to stay active during the pandemic. The highest proportion of sedentary behaviour was during school hours on a school day, while on a non-school day it peaked from late afternoon to evening. During free time, participants engaged in screen time much more for leisure than educational purposes, with the most favourite content being action games. The most used device was the smartphone, which was mostly used in the bedroom in a solitary context. Non-screen-based sedentary behaviour was consistently low. Interviews suggested that the high amount of screen time seemed to be influenced by educational demands, devices and internet availability, more opportunities for screen time, lack of parental control, social facilitators from friends and family members, phone notifications, and emotional state. Using digital platforms may be beneficial to shift some screen-based sedentary behaviour to ‘screen-based’ or ‘screen-prompted’ physical activity.

## Supplementary Information

**Additional file 1:** **Table S1**. Accelerometer cut points for children and adolescents.

**Additional file 2:** **Table S2**: Coding Guideline for Automated Wearable Camera Data.

**Additional file 3**: Interview Guideline.

**Additional file 4:** **Table S4.1**: Purpose of activity based on camera data; **Table S4.2**: Physical setting of activity based on camera data; **Table S4.3**: Social context, environment, and interaction based on camera data.

## Data Availability

The datasets used and/or analysed during the current study are available from the corresponding author on reasonable request.

## Abbreviations

COVID-19: Severe acute respiratory syndrome coronavirus 2 (SARS-CoV-2)
LMICs: Low- and middle-income countries
BMI: Body mass index
SB: Sedentary behaviour
PA: Physical activity
LPA: Light physical activity
MPA: Moderate physical activity
VPA: Vigorous physical activity
SD: Standard deviation

## References

[1] WHO. COVID-19—a global pandemic, What do we know about SARS-CoV-2 and COVID-19?2020 6 January 2021 [cited 2021 6 January]:[18 p.]. Available from: https://www.who.int/docs/default-source/coronaviruse/risk-comms-updates/update-28-covid-19-what-we-know-may-2020.pdf?sfvrsn=ed6e286c_2.

[2] WHO. Listings of WHO’s response to COVID-19 2020 [cited 2022 7 August]. Available from: https://www.who.int/news/item/29-06-2020-covidtimeline.

[3] UNICEF. UNICEF Education COVID-19 Case Study: Indonesia—Contextualizing global COVID-19 guidelines to the local context: UNICEF; 2020 [cited 2022 7 August]. Available from: https://reliefweb.int/report/indonesia/unicef-education-covid-19-case-study-indonesia-contextualizing-global-covid-19.

[4] Storen R, Corrigan N. COVID-19: a chronology of state and territory government announcements (up until 30 June 2020): Parliament of Australia; 2020 [cited 2022 7 August]. Available from: https://www.aph.gov.au/About_Parliament/Parliamentary_Departments/Parliamentary_Library/pubs/rp/rp2021/Chronologies/COVID-19StateTerritoryGovernmentAnnouncements.

[5] Stockwell S, Trott M, Tully M, Shin J, Barnett Y, Butler L, et al. Changes in physical activity and sedentary behaviours from before to during the COVID-19 pandemic lockdown: a systematic review. BMJ Open Sport Exerc Med. 2021;7(1): e000960. 10.1136/bmjsem-2020-000960 PMC7852071 34192010

[6] Yomoda K, Kurita S. Influence of social distancing during the COVID-19 pandemic on physical activity in children: a scoping review of the literature. J Exerc Sci Fit. 2021;19(3):195–203. 34135976 10.1016/j.jesf.2021.04.002 PMC8164031

[7] Zenic N, Taiar R, Gilic B, Blazevic M, Maric D, Pojskic H, et al. Levels and changes of physical activity in adolescents during the COVID-19 pandemic: contextualizing urban vs. rural living environment. Appl Sci. 2020;10(11):3997.

[8] Xiang M, Zhang Z, Kuwahara K. Impact of COVID-19 pandemic on children and adolescents’ lifestyle behavior larger than expected. Prog Cardiovasc Dis. 2020;63(4):531–2. 32360513 10.1016/j.pcad.2020.04.013 PMC7190470

[9] Bates LC, Zieff G, Stanford K, Moore JB, Kerr ZY, Hanson ED, et al. COVID-19 impact on behaviors across the 24-hour day in children and adolescents: physical activity, sedentary behavior, and sleep. Children (Basel). 2020;7(9):138. 32947805 10.3390/children7090138 PMC7552759

[10] Dunton GF, Do B, Wang SD. Early effects of the COVID-19 pandemic on physical activity and sedentary behavior in children living in the U.S. BMC Public Health. 2020;20(1351). 10.1186/s12889-020-09429-3 PMC7472405 32887592

[11] McCormack GR, Doyle-Baker PK, Petersen JA, Ghoneim D. Parent anxiety and perceptions of their child’s physical activity and sedentary behaviour during the COVID-19 pandemic in Canada. Prev Med Rep. 2020;20: 101275. 33282637 10.1016/j.pmedr.2020.101275 PMC7708797

[12] Ng K, Cooper J, McHale F, Clifford J, Woods C. Barriers and facilitators to changes in adolescent physical activity during COVID-19. BMJ Open Sport Exerc Med. 2020;6(1): e000919. 10.1136/bmjsem-2020-000919 PMC7673110 33262893

[13] Gilic B, Ostojic L, Corluka M, Volaric T, Sekulic D. Contextualizing parental/familial influence on physical activity in adolescents before and during COVID-19 pandemic: a prospective analysis. Children (Basel). 2020;7(9). 10.3390/children7090125 PMC7552694 32899277

[14] Mitra R, Moore SA, Gillespie M, Faulkner G, Vanderloo LM, Chulak-Bozzer T, et al. Healthy movement behaviours in children and youth during the COVID-19 pandemic: exploring the role of the neighbourhood environment. Health Place. 2020;65: 102418. 32871499 10.1016/j.healthplace.2020.102418 PMC7455528

[15] Cheval B, Sivaramakrishnan H, Maltagliati S, Fessler L, Forestier C, Sarrazin P, et al. Relationships between changes in self-reported physical activity, sedentary behaviour and health during the coronavirus (COVID-19) pandemic in France and Switzerland. J Sports Sci. 2020;39(6):699–704. 33118469 10.1080/02640414.2020.1841396

[16] Barnett TA, Kelly AS, Young DR, Perry CK, Pratt CA, Edwards NM, et al. Sedentary behaviors in today’s youth: approaches to the prevention and management of childhood obesity: a scientific statement from the American Heart Association. Circulation. 2018;138(11). 10.1161/CIR.0000000000000591 30354382

[17] Andriyani FD, Biddle SJH, Priambadha AA, Thomas G, De Cocker K. Physical activity and sedentary behaviour of female adolescents in Indonesia: a multi-method study on duration, pattern and context. J Exerc Sci Fit. 2022;20(2):128–39. 35308068 10.1016/j.jesf.2022.02.002 PMC8899402

[18] Thomas G, Bennie JA, De Cocker K, Andriyani FD, Booker B, Biddle SJH. Using wearable cameras to categorize the type and context of screen-based behaviors among adolescents: observational study. JMIR Pediatr Parent. 2022;5(1): e28208. 35311672 10.2196/28208 PMC8981006

[19] Guthold R, Stevens GA, Riley LM, Bull FC. Global trends in insufficient physical activity among adolescents: a pooled analysis of 298 population-based surveys with 1·6 million participants. Lancet Child Adolesc Health. 2020;4(1):23–35. 31761562 10.1016/S2352-4642(19)30323-2 PMC6919336

[20] Aubert S, Brazo-Sayavera J, Gonzalez SA, Janssen I, Manyanga T, Oyeyemi AL, et al. Global prevalence of physical activity for children and adolescents; inconsistencies, research gaps, and recommendations: a narrative review. Int J Behav Nutr Phys Act. 2021;18(1):81. 34187486 10.1186/s12966-021-01155-2 PMC8243483

[21] Thomas G, Bennie JA, De Cocker K, Ireland MJ, Biddle SJH. Screen-based behaviors in Australian adolescents: longitudinal trends from a 4-year follow-up study. Prev Med. 2020;141: 106258. 33022322 10.1016/j.ypmed.2020.106258

[22] Thomas G, Bennie JA, De Cocker K, Biddle SJH. Exploring contemporary screen time in Australian adolescents: a qualitative study. Health Promot J Austr. 2020;32(S2):238–47. 10.1002/hpja.440 33185908

[23] Andriyani FD, Biddle SJH, De Cocker K. Adolescents’ physical activity and sedentary behaviour in Indonesia during the COVID-19 pandemic: a qualitative study of mothers’ perspectives. BMC Public Health. 2021;21(1):1864 10.1186/s12889-021-11931-1 PMC8519321 34654384

[24] Hallal PC, Bauman AE, Heath GW, Kohl HW, Lee I-M, Pratt M. Physical activity: more of the same is not enough. The Lancet. 2012;380(9838):190–1. 10.1016/S0140-6736(12)61027-7 22818932

[25] Andriyani FD, Biddle SJH, Arovah NI, Cocker K. Physical activity and sedentary behavior research in Indonesian youth: a scoping review. Int J Environ Res Public Health. 2020;17(20):7665. 33096653 10.3390/ijerph17207665 PMC7593924

[26] Crowe S, Cresswell K, Robertson A, Huby G, Avery A, Sheikh A. The case study approach. BMC Med Res Methodol. 2011;11:100. 21707982 10.1186/1471-2288-11-100 PMC3141799

[27] Creswell JW. Mixed-method research: Introduction and application. Handbook of educational policy: Elsevier; 1999. p. 455–72.

[28] Centers for Disease Control and Prevention US. BMI calculator for child and teen: Centers for Disease Control and Prevention; 2021 [cited 2022. Available from: https://www.cdc.gov/healthyweight/bmi/result.html?&method=metric&gender=m&age_y=14&age_m=10&hcm=173&wkg=63.8.

[29] Donaldson SC, Montoye AH, Tuttle MS, Kaminsky LA. Variability of objectively measured sedentary behavior. Med Sci Sports Exerc. 2016;48(4):755–61. 26606270 10.1249/MSS.0000000000000828

[30] Chandler JL, Brazendale K, Beets MW, Mealing BA. Classification of physical activity intensities using a wrist-worn accelerometer in 8–12-year-old children. Pediatr Obes. 2015;11(2):120–7. 25893950 10.1111/ijpo.12033

[31] Chandler J, Beets M, Saint-Maurice P, Weaver R, Cliff D, Drenowatz C, et al. Wrist-based accelerometer cut-points to identify sedentary time in 5(-)11-year-old children. Children (Basel). 2018;5(10). 10.3390/children5100137 PMC6210293 30261646

[32] Scott JJ, Rowlands AV, Cliff DP, Morgan PJ, Plotnikoff RC, Lubans DR. Comparability and feasibility of wrist- and hip-worn accelerometers in free-living adolescents. J Sci Med Sport. 2017;20(12):1101–6. 28501418 10.1016/j.jsams.2017.04.017

[33] Fairclough SJ, Noonan R, Rowlands AV, Van Hees V, Knowles Z, Boddy LM. Wear compliance and activity in children wearing wrist- and hip-mounted accelerometers. Med Sci Sports Exerc. 2016;48(2):245–53. 26375253 10.1249/MSS.0000000000000771

[34] Migueles JH, Cadenas-Sanchez C, Ekelund U, Delisle Nystrom C, Mora-Gonzalez J, Lof M, et al. Accelerometer data collection and processing criteria to assess physical activity and other outcomes: a systematic review and practical considerations. Sports Med. 2017;47(9):1821–45. 28303543 10.1007/s40279-017-0716-0 PMC6231536

[35] Kelly P, Marshall SJ, Badland H, Kerr J, Oliver M, Doherty AR, et al. An ethical framework for automated, wearable cameras in health behavior research. Am J Prev Med. 2013;44(3):314–9. 23415131 10.1016/j.amepre.2012.11.006

[36] Sweetser P, Johnson D, Ozdowska A, Wyeth P. Active versus passive screen time for young children. Aust J Early Child. 2012;37(4).

[37] Syed M, Nelson SC. Guidelines for establishing reliability when coding narrative data. Emerg Adulthood. 2015;3(6):375–87.

[38] Butte NF, Watson KB, Ridley K, Zakeri IF, McMurray RG, Pfeiffer KA, et al. A youth compendium of physical activities: activity codes and metabolic intensities. Med Sci Sports Exerc. 2018;50(2):246–56. 28938248 10.1249/MSS.0000000000001430 PMC5768467

[39] Braun V, Clarke V. Reflecting on reflexive thematic analysis. Qual Res Sport Exerc Health. 2019;11(4):589–97.

[40] Braun V, Clarke V. Thematic analysis: a reflexive approach2020 8 January 2021 [cited 2021 8 January]. Available from: https://www.psych.auckland.ac.nz/en/about/thematic-analysis.html.

[41] WHO. COVID-19—a global pandemic: What do we know about SARS-CoV-2 and COVID-19?2020 3 February 2021 [cited 2021 3 February]. Available from: https://www.who.int/docs/default-source/coronaviruse/risk-comms-updates/update-28-covid-19-what-we-know-may-2020.pdf?sfvrsn=ed6e286c_2.

[42] Pemerintah Daerah DIY. Sri Sultan keluarkan kebijakan belajar di rumah2020 3 February 2021 [cited 2021 3 February]. Available from: https://jogjaprov.go.id/berita/detail/8591-sri-sultan-keluarkan-kebijakan-belajar-di-rumah.

[43] Hakim L. DIY memperpanjang tanggap darurat COVID-19 sampai 30 November: ANTARA; 2020 [cited 2022 12 August]. Available from: https://jogja.antaranews.com/berita/459061/diy-memperpanjang-tanggap-darurat-covid-19-sampai-30-november.

[44] Moore SA, Faulkner G, Rhodes RE, Brussoni M, Chulak-Bozzer T, Ferguson LJ, et al. Impact of the COVID-19 virus outbreak on movement and play behaviours of Canadian children and youth: a national survey. Int J Behav Nutr Phys Act. 2020;17(1):85. 32631350 10.1186/s12966-020-00987-8 PMC7336091

[45] Guerrero MD, Vanderloo LM, Rhodes RE, Faulkner G, Moore SA, Tremblay MS. Canadian children’s and youth’s adherence to the 24-h movement guidelines during the COVID-19 pandemic: a decision tree analysis. J Sport Health Sci. 2020;9(4):313–21. 32525098 10.1016/j.jshs.2020.06.005 PMC7276134

[46] Belton S, O’Brien W, Issartel J, McGrane B, Powell D. Where does the time go? Patterns of physical activity in adolescent youth. J Sci Med Sport. 2016;19(11):921–5. 26897391 10.1016/j.jsams.2016.01.008

[47] Poitras VJ, Gray CE, Borghese MM, Carson V, Chaput JP, Janssen I, et al. Systematic review of the relationships between objectively measured physical activity and health indicators in school-aged children and youth. Appl Physiol Nutr Metab. 2016;41(6 Suppl 3):S197-239. 27306431 10.1139/apnm-2015-0663

[48] Kandola A, Lewis G, Osborn DPJ, Stubbs B, Hayes JF. Depressive symptoms and objectively measured physical activity and sedentary behaviour throughout adolescence: a prospective cohort study. The Lancet Psychiatry. 2020;7(3):262–71. 32059797 10.1016/S2215-0366(20)30034-1 PMC7033559

[49] Chambonniere C, Lambert C, Fearnbach N, Tardieu M, Fillon A, Genin P, et al. Effect of the COVID-19 lockdown on physical activity and sedentary behaviors in French children and adolescents: new results from the ONAPS national survey. Eur J Integr Med. 2021;43: 101308. 33584872 10.1016/j.eujim.2021.101308 PMC7871771

[50] Andriyani FD, Biddle SJH, De Cocker K. Adolescents’ physical activity and sedentary behaviour in Indonesia during the COVID-19 pandemic: a qualitative study of mothers’ perspectives. BMC Public Health. 2021;21(1):1864. 34654384 10.1186/s12889-021-11931-1 PMC8519321

[51] Jago R, Brockman R, Fox KR, Cartwright K, Page AS, Thompson JL. Friendship groups and physical activity: qualitative findings on how physical activity is initiated and maintained among 10–11 year old children. Int J Behav Nutr Phys Act. 2009;6:4. 19138411 10.1186/1479-5868-6-4 PMC2631002

[52] Garcia JM, Sirard JR, Deutsch NL, Weltman A. The influence of friends and psychosocial factors on physical activity and screen time behavior in adolescents: a mixed-methods analysis. J Behav Med. 2016;39(4):610–23. 27055818 10.1007/s10865-016-9738-6

[53] Thompson AM, Rehman LA, Humbert ML. Factors influencing the physically active leisure of children and youth: a qualitative study. Leis Sci. 2005;27(5):421–38.

[54] Moor E. 50 Exercises and activities for at-home P.E. 2020 [cited 2022 24 September]. Available from: https://teacherblog.evan-moor.com/2020/11/03/50-exercises-and-activities-for-at-home-p-e/.

[55] Landers B. How to stay active at home: PE at home resources n.d. [cited 2022 24 September]. Available from: https://www.thepespecialist.com/peathome/.

[56] Atkin AJ, Gorely T, Biddle SJH, Marshall SJ, Cameron N. Critical Hours: Physical Activity and Sedentary Behavior of Adolescents after School. Pediatr Exerc Sci. 2008;20(4):446–56. 19168921 10.1123/pes.20.4.446

[57] Toh SH, Howie EK, Coenen P, Straker LM. "From the moment I wake up I will use it...every day, very hour": a qualitative study on the patterns of adolescents' mobile touch screen device use from adolescent and parent perspectives. BMC Pediatr. 2019;19(1):30. 10.1186/s12887-019-1399-5 PMC6346550 30678720

[58] Granich J, Rosenberg M, Knuiman M, Timperio A. Understanding children’s sedentary behaviour: a qualitative study of the family home environment. Health Educ Res. 2010;25(2):199–210. 18502732 10.1093/her/cyn025

[59] Tremblay MS, LeBlanc AG, Kho ME, Saunders TJ, Larouche R, Colley RC, et al. Systematic review of sedentary behaviour and health indicators in school-aged children and youth. Int J Behav Nutr Phys Act. 2011;8(98):1–22. 21936895 10.1186/1479-5868-8-98 PMC3186735

[60] Carson V, Hunter S, Kuzik N, Gray CE, Poitras VJ, Chaput JP, et al. Systematic review of sedentary behaviour and health indicators in school-aged children and youth: an update. Appl Physiol Nutr Metab. 2016;41(6 Suppl 3):S240–65. 27306432 10.1139/apnm-2015-0630

[61] Chaput JP, Willumsen J, Bull F, Chou R, Ekelund U, Firth J, et al. 2020 WHO guidelines on physical activity and sedentary behaviour for children and adolescents aged 5–17 years: summary of the evidence. Int J Behav Nutr Phys Act. 2020;17(1):141. 33239009 10.1186/s12966-020-01037-z PMC7691077

[62] Parks M, Solmon M, Lee A. Understanding classroom teachers’ perceptions of integrating physical activity: a collective efficacy perspective. J Res Child Educ. 2007;21(3):316–28.

[63] Reeves E, Miller S, Chavez C. Movement and learning: integrating physical activity into the classroom. Kappa Delta Pi Record. 2016;52(3):116–20.

[64] Allen B, Waterman H. Stages of adolescence2019 3 September 2021. Available from: https://www.healthychildren.org/English/ages-stages/teen/Pages/Stages-of-Adolescence.aspx.

[65] Institute of Medicine NRC. The science of adolescent risk-taking The United States of America: The National Academy of Sciences; 2011 [cited 2021 26 February]. Available from: https://www.ncbi.nlm.nih.gov/books/NBK53418/.

[66] Sanders T, Parker PD, Del Pozo-Cruz B, Noetel M, Lonsdale C. Type of screen time moderates effects on outcomes in 4013 children: evidence from the Longitudinal Study of Australian Children. Int J Behav Nutr Phys Act. 2019;16(1):117. 31783878 10.1186/s12966-019-0881-7 PMC6884886

[67] King DL, Delfabbro PH, Billieux J, Potenza MN. Problematic online gaming and the COVID-19 pandemic. J Behav Addict. 2020;9(2):184–6. 32352927 10.1556/2006.2020.00016 PMC8939428

[68] Parker K, Uddin R, Ridgers ND, Brown H, Veitch J, Salmon J, et al. The use of digital platforms for adults’ and adolescents’ physical activity during the COVID-19 pandemic (our life at home): survey study. J Med Internet Res. 2021;23(2): e23389. 33481759 10.2196/23389 PMC7857525

