# Supplementary material for: Physical activity and sedentary behaviour of male adolescents in Indonesia during the COVID-19 pandemic: a mixed-method case study using accelerometers, automated wearable cameras, diaries, and interviews
Source: J Act Sedentary Sleep Behav. 2023 Mar 1;2:5. doi: 10.1186/s44167-022-00014-0 (PMC9974395; doi:10.1186/s44167-022-00014-0)
Supplement: Supplementary file 1 — Additional file 1: Table S1. Accelerometer cut points for children and adolescents. [file 44167_2022_14_MOESM1_ESM.docx]

**Physical activity and sedentary behaviour of male adolescents in Indonesia during the COVID-19 pandemic: A mixed-method study using accelerometers, automated wearable cameras, diaries, and interviews**

Fitria Dwi Andriyani, Katrien De Cocker, Aprida Agung Priambadha, Stuart J.H. Biddle

**Additional File 1**

Table S1. Accelerometer cut points for children and adolescents based on Chandler et al. [29]

| **Axis** | **Intensity Category** | **Counts per 60 seconds** |
| --- | --- | --- |
| Vector Magnitude | Sedentary | <3660 |
|  | Light physical activity | 3660-9815 |
|  | Moderate physical activity | 9816-23628 |
|  | Vigorous physical activity | >23628 |
