# Supplementary material for: Physical activity and sedentary behaviour of male adolescents in Indonesia during the COVID-19 pandemic: a mixed-method case study using accelerometers, automated wearable cameras, diaries, and interviews
Source: J Act Sedentary Sleep Behav. 2023 Mar 1;2:5. doi: 10.1186/s44167-022-00014-0 (PMC9974395; doi:10.1186/s44167-022-00014-0)
Supplement: Supplementary file 3 — Additional file 3: Interview Guideline. [file 44167_2022_14_MOESM3_ESM.docx]

**Physical activity and sedentary behaviour of male adolescents in Indonesia during the COVID-19 pandemic: A mixed-method study using accelerometers, automated wearable cameras, diaries, and interviews**

Fitria Dwi Andriyani, Katrien De Cocker, Aprida Agung Priambadha, Stuart J.H. Biddle

**Additional File 3. Interview Guideline**

| **Question** |
| --- |

Name :

Date of interview :

**Tell the outline of the interview**

**Tell the definition of sedentary behaviour and examples of both screen- and non-screen-based sedentary behaviour**

**Compare behaviour before and during the COVID-19 pandemic**

1. **Motivational, purposes, contextual reasons, and rules for sedentary behaviour**
2. **Screen-based sedentary behaviour in leisure time**
3. What screen-based device do you **use** most? Why? What for?

**In a day, which purpose do you do most frequently?**

1. When do you use screen-based device most? Why?
2. Where do you use screen-based device most? Why?
3. With who do you use screen-based device most?
4. What triggers you to start using screen-based device?
5. What triggers you to stop using screen-based device?
6. Is there any rule about this at your home? If yes, does the rule affects the way you use scrren-based device? What do you think about having rules?
7. What kind of support do you get to control your recreational screen time?
8. What kind of barriers do you feel to limit your recreational screen time?
9. **Non-screen-based sedentary behaviour in leisure time**
10. What non-screen-based sedentary behaviour do you do most? Why? What for?
11. When do you do non-screen-based sedentary behaviour most? Why?
12. Where do you do non-screen-based sedentary behaviour most? Why?
13. With who do you do non-screen-based sedentary behaviour most?
14. What triggers you to start doing non-screen-based sedentary behaviour?
15. What triggers you to stop doing non-screen-based sedentary behaviour?
16. Is there any rule about this at your home? If yes, does the rule affects the way you use scrren-based device? What do you think about having rules?
17. What kind of support do you get for controlling your non-screen-based sedentary behaviour?
18. What kind of barriers do you feel to limit your non-screen-based sedentary behaviour?
19. **The perceived value of screen-based sedentary behaviour**

Internationally, there is a recommendation to limit recreational screen time to be maximum 2 hours

1. What do you feel about your recreational screen time? Do you think it is too much or about right?
2. What are the benefits of screen time for you?
3. What are the effects of screen time for you?
4. Do you think screen time give more positive or negative effects for you? Why?
5. What do you do to get positive effects from screen time?
6. What do you do to protect yourself the negative effects from screen time? (e.g., accessing inappropriate video or games)
7. **Why do you decided to participate in this study? How do you feel when taking part in this study? Is there any problem during the data collection process?**
