# Supplementary material for: Physical activity and sedentary behaviour of male adolescents in Indonesia during the COVID-19 pandemic: a mixed-method case study using accelerometers, automated wearable cameras, diaries, and interviews
Source: J Act Sedentary Sleep Behav. 2023 Mar 1;2:5. doi: 10.1186/s44167-022-00014-0 (PMC9974395; doi:10.1186/s44167-022-00014-0)
Supplement: Supplementary file 4 — Additional file 4: Table S4.1: Purpose of activity based on camera data; Table S4.2: Physical setting of activity based on camera data; Table S4.3: Social context, environment, and interaction based on camera data. [file 44167_2022_14_MOESM4_ESM.docx]

**Physical activity and sedentary behaviour of male adolescents in Indonesia during the COVID-19 pandemic: A mixed-method study using accelerometers, automated wearable cameras, diaries, and interviews**

Fitria Dwi Andriyani, Katrien De Cocker, Aprida Agung Priambadha, Stuart J.H. Biddle

**Additional File 4**

| **Table S4.1. Purpose of activity based on camera data** |  | | |  | | |  |  |  |
| --- | --- | --- | --- | --- | --- | --- | --- | --- | --- |
| **Purpose** | ***n* of images** | | | **%** | | |  |  |  |
| **Movement behaviours** |  | | |  | | |  |  |  |
| *Leisure* | 139 | | | 8 | | |  |  |  |
| *Transport* | 20 | | | 1 | | |  |  |  |
| *Social* | 69 | | | 4 | | |  |  |  |
| *Personal Care* | 291 | | | 18 | | |  |  |  |
| *Domestic* | 87 | | | 5 | | |  |  |  |
| *Other* | 117 | | | 7 | | |  |  |  |
| *Unclassifiable* | 932 | | | 56 | | |  |  |  |
| **SUM** | **1655** | | | **100** | | |  |  |  |
| **Screen-based Sedentary Behaviour** |  | | |  | | |  |  |  |
| **Portable Device: Mobile Phone (smartphone)** |  | | |  | | |  |  |  |
| *Leisure* | 10329 | | | 63 | | |  |  |  |
| *Other* | 7 | | | 0 | | |  |  |  |
| *Unclassifiable* | 3310 | | | 20 | | |  |  |  |
| *Social* | 2044 | | | 12 | | |  |  |  |
| *Educational* | 788 | | | 5 | | |  |  |  |
| **SUM** | **16478** | | | **100** | | |  |  |  |
| **Portable Device: Laptop** |  | | |  | | |  |  |  |
| *Unclassifiable* | 372 | | | 26 | | |  |  |  |
| *Leisure* | 1043 | | | 74 | | |  |  |  |
| **SUM** | **1415** | | | **100** | | |  |  |  |
| **Non-Portable Device: Television** |  | | |  | | |  |  |  |
| *Leisure* | 3732 | | | 100 | | |  |  |  |
| **Non-Portable Device: Television for gaming** |  | | |  | | |  |  |  |
| *Leisure* | 506 | | | 100 | | |  |  |  |
| **Non-screen-based Sedentary Behaviour** |  | | |  | | |  |  |  |
| *Educational* | 350 | | | 13 | | |  |  |  |
| *Transport* | 197 | | | 7 | | |  |  |  |
| *Other* | 169 | | | 6 | | |  |  |  |
| *Unclassifiable* | 725 | | | 27 | | |  |  |  |
| *Personal Care* | 485 | | | 18 | | |  |  |  |
| *Social* | 701 | | | 27 | | |  |  |  |
| *Domestic* | 10 | | | 0 | | |  |  |  |
| **SUM** | **2637** | | | **100** | | |  |  |  |
| **Table S4.2. Physical setting of activity based on camera data** | |  | | |  | | |  |  |
| **Physical Setting** | | ***n* of images** | | | **%** | | |  |  |
| **Movement Behaviours** | |  | | |  | | |  |  |
| *Home: Bedroom* | | 161 | | | 10 | | |  |  |
| *Home: Living room* | | 108 | | | 7 | | |  |  |
| *Home: Outside* | | 311 | | | 19 | | |  |  |
| *Home: Other (e.g., Office, bathroom)* | | 116 | | | 7 | | |  |  |
| *Home: Kitchen/Dining Room* | | 141 | | | 9 | | |  |  |
| *Home: Unclassifiable* | | 443 | | | 27 | | |  |  |
| *Public: Street* | | 127 | | | 8 | | |  |  |
| *Public: Retail* | | 15 | | | 1 | | |  |  |
| *Public: Recreation space* | | 192 | | | 12 | | |  |  |
| *Public: Food Retail* | | 41 | | | 2 | | |  |  |
| **SUM** | | **1655** | | | **100** | | |  |  |
| **Screen-based Sedentary Behaviour** | |  | | |  | | |  |  |
| **Portable Device: Mobile phone (smartphone)** | | | | |  | | |  |  |
| *Home: Bedroom* | | 14534 | | | 88 | | |  |  |
| *Home: Living room* | | 595 | | | 4 | | |  |  |
| *Home: Outside* | | 249 | | | 2 | | |  |  |
| *Home: Other (e.g., Office, bathroom)* | | 188 | | | 1 | | |  |  |
| *Home: Unclassifiable* | | 68 | | | 0 | | |  |  |
| *Public: Street* | | 844 | | | 5 | | |  |  |
| **SUM** | | **16478** | | | **100** | | |  |  |
| **Portable: Laptop Computer** | |  | | |  | | |  |  |
| ***Home: Bedroom*** | | **1415** | | | **100** | | |  |  |
| **Non-Portable Device: Television** | |  | | |  | | |  |  |
| *Home: Bedroom* | | 3561 | | | 95 | | |  |  |
| *Home: Living room* | | 149 | | | 4 | | |  |  |
| *Home: Other (e.g., Office, bathroom)* | | 22 | | | 1 | | |  |  |
| **SUM** | | **3732** | | | **100** | | |  |  |
| **Non-Portable Device: Television for gaming** | |  | | |  | | |  |  |
| ***Home: Bedroom*** | | **506** | | | **100** | | |  |  |
| **Non-screen-based Sedentary Behaviour** | |  | | |  | | |  |  |
| *Home: Bedroom* | | 853 | | | 32 | | |  |  |
| *Home: Living room* | | 595 | | | 23 | | |  |  |
| *Home: Outside* | | 288 | | | 11 | | |  |  |
| *Home: Other (e.g., Office, bathroom)* | | 295 | | | 11 | | |  |  |
| *Home: Kitchen/Dining Room* | | 10 | | | 0 | | |  |  |
| *Home: Unclassifiable* | | 206 | | | 8 | | |  |  |
| *Transport: Private Transport* | | 111 | | | 4 | | |  |  |
| *Public: Recreation space* | | 4 | | | 0 | | |  |  |
| *Public: Food Retail* | | 8 | | | 0 | | |  |  |
| *Public: Retail* | | 7 | | | 0 | | |  |  |
| *Public: Street* | | 260 | | | 10 | | |  |  |
| **SUM** | | **2637** | | | **100** | | |  |  |
| **Table S4.3. Social context, environment, and interaction based on camera data** | | | |  | | |  | | |
| **Variable** | | | | ***n* of images** | | | **%** | | |
| **Movement Behaviours** | | | |  | | |  | | |
| **Social Context** | | | |  | | |  | | |
| *Alone* | | | | 1028 | | | 62 | | |
| *Direct social engagement* | | | | 354 | | | 21 | | |
| *Social environment but no interaction* | | | | 273 | | | 16 | | |
| **Social Environment** | | | |  | | |  | | |
| *Alone (i.e., none)* | | | | 1028 | | | 62 | | |
| *Adult&child* | | | | 136 | | | 8 | | |
| *Child* | | | | 256 | | | 15 | | |
| *Adult* | | | | 136 | | | 8 | | |
| *Unclassifiable* | | | | 99 | | | 6 | | |
| **Social Interaction** | | | |  | | |  | | |
| *None* | | | | 1293 | | | 78 | | |
| *Conversation* | | | | 152 | | | 9 | | |
| *Co-viewing* | | | | 13 | | | 1 | | |
| *Co-participating* | | | | 34 | | | 2 | | |
| *Other* | | | | 140 | | | 8 | | |
| *Unclassifiable* | | | | 20 | | | 1 | | |
| **Screen-based Sedentary Behaviour** | | | |  | | |  | | |
| **Portable Device>Mobile phone (smartphone)** | | | |  | | |  | | |
| **Social Context** | | | |  | | |  | | |
| *Alone* | | | | 12995 | | | 79 | | |
| *Direct social engagement* | | | | 1331 | | | 8 | | |
| *Social environment but no interaction* | | | | 2152 | | | 13 | | |
| **Social Environment** | | | |  | | |  | | |
| *Alone (i.e., none)* | | | | 12998 | | | 79 | | |
| *Adult&child* | | | | 950 | | | 6 | | |
| *Child* | | | | 1550 | | | 9 | | |
| *Adult* | | | | 970 | | | 6 | | |
| *Unclassifiable* | | | | 10 | | | 0 | | |
| **Social Interaction** | | | |  | | |  | | |
| *None* | | | | 14449 | | | 88 | | |
| *Conversation* | | | | 43 | | | 0 | | |
| *Background* | | | | 582 | | | 4 | | |
| *Co-viewing* | | | | 1198 | | | 7 | | |
| *Unclassifiable* | | | | 206 | | | 1 | | |
| **Portable >Laptop Computer** | | | |  | | |  | | |
| **Social Context** | | | |  | | |  | | |
| *Alone* | | | | 1415 | | | 100 | | |
| **Social Environment** | | | |  | | |  | | |
| *Alone (i.e., none)* | | | | 1415 | | | 100 | | |
| **Social Interaction** | | | |  | | |  | | |
| *None* | | | | 1415 | | | 100 | | |
| **Non-Portable Device: Television** | | | |  | | |  | | |
| **Social Context** | | | |  | | |  | | |
| *Alone* | | | | 1995 | | | 53 | | |
| *Direct social engagement* | | | | 1495 | | | 40 | | |
| *Social environment but no interaction* | | | | 242 | | | 6 | | |
| **Social Environment** | | | |  | | |  | | |
| *Alone (i.e., none)* | | | | 1995 | | | 53 | | |
| *Adult&child* | | | | 466 | | | 12 | | |
| *Child* | | | | 457 | | | 12 | | |
| *Adult* | | | | 748 | | | 20 | | |
| *Unclassifiable* | | | | 66 | | | 2 | | |
| **Social Interaction** | | | |  | | |  | | |
| *None* | | | | 2015 | | | 54 | | |
| *Conversation* | | | | 6 | | | 0 | | |
| *Background* | | | | 12 | | | 0 | | |
| *Co-viewing* | | | | 1677 | | | 45 | | |
| *Unclassifiable* | | | | 22 | | | 1 | | |
| **Non-Portable Device: Television for gaming** | | | |  | | |  | | |
| **Social Context** | | | |  | | |  | | |
| *Alone* | | | | 15 | | | 3 | | |
| *Direct social engagement* | | | | 491 | | | 97 | | |
| **Social Environment** | | | |  | | |  | | |
| *Alone (i.e., none)* | | | | 15 | | | 3 | | |
| *Adult* | | | | 153 | | | 30 | | |
| *Unclassifiable* | | | | 338 | | | 67 | | |
| **Social Interaction** | | | |  | | |  | | |
| *None* | | | | 15 | | | 3 | | |
| *Co-participating* | | | | 491 | | | 97 | | |
| **Non-screen-based Sedentary Behaviour** | | | |  | | |  | | |
| **Social Context** | | | |  | | |  | | |
| *Alone* | | | | 1380 | | | 52 | | |
| *Direct social engagement* | | | | 1131 | | | 43 | | |
| *Social environment but no interaction* | | | | 126 | | | 5 | | |
| **Social Environment** | | | |  | | |  | | |
| *Alone (i.e., none)* | | | | 1380 | | | 52 | | |
| *Adult&child* | | | | 152 | | | 6 | | |
| *Child* | | | | 951 | | | 36 | | |
| *Adult* | | | | 141 | | | 5 | | |
| *Unclassifiable* | | | | 13 | | | 0 | | |
| **Social Interaction** | | | |  | | |  | | |
| *None* | | | | 1430 | | | 54 | | |
| *Conversation* | | | | 867 | | | 33 | | |
| *Background* | | | | 74 | | | 3 | | |
| *Other* | | | | 174 | | | 7 | | |
| *Unclassifiable* | | | | 92 | | | 3 | | |
